# Contraceptive use and decision-making in Guéra, Chad: a mixed-methods study

**DOI:** 10.3389/fgwh.2025.1516757

**Published:** 2025-06-12

**Authors:** Vincent de Paul Allambademel, Mahamat Abdelaziz, Natalie Andrasko, Bongo Goumbo, Robert Madjigoto, Alexis Ngarmbatedjimal, Solal Noubadjim, Salomon Tamira, Theodora Varelis, Katchebe Vourbane, Sara E. Casey

**Affiliations:** ^1^ Laboratoire de Sociologie, d’Anthropologie et des Etudes Africaines (LASA), Department of Sociology, College of Humanities and Social Sciences, University of N'Djamena, N'Djamena, Chad; ^2^ RAISE Initiative, Heilbrunn Department of Population and Family Health, Mailman School of Public Health, Columbia University, New York, NY, United States; ^3^ International Rescue Committee, N'Djamena, Chad

**Keywords:** contraception, Chad, sexual and reproductive health, mixed methods, decision-making

## Abstract

**Background:**

Chad has one of the highest maternal mortality ratios in the world, and low modern contraceptive prevalence. Understanding the barriers and influences on women's decision-making around contraceptive use is critical to reducing the unmet contraceptive need, and thus maternal mortality.

**Methods:**

A mixed-methods study was conducted in three districts of Guéra province, Chad, including a longitudinal survey of women, focus group discussions with male and female users and non-users of modern contraceptives, and in-depth interviews with midwives, community health workers, and community leaders.

**Results:**

Survey participants showed increased knowledge of modern contraceptive methods, and 20.5% reported current modern contraceptive use at endline. Participants described multiple reasons for contraceptive non-use, including that it contradicts with their religious beliefs, community stigma and widespread misconceptions, particularly around long-acting reversible contraceptives. Husbands played a large role in a couple's contraceptive decision-making, as either a major facilitator or barrier depending on the individual.

**Discussion:**

Overall, the study's findings suggest that participant awareness of modern contraceptive methods increased. Contraceptive use was more than twice as high as the provincial rate. Given the dominance of injectables within our sample, programs should explore introducing and scaling up community-based distribution of the self-injectable contraceptive (DMPA-SC). The findings highlight the need for more diverse and tailored stigma reduction interventions in the community to promote awareness and reduce misconceptions of modern contraceptives among key groups of people, including men, couples, and religious and other community leaders. Values clarification and attitude training should be considered for all cadres of providers to promote non-discrimination and equity in contraceptive service provision.

## Introduction

Women's access to high-quality, equitable, and rights-based sexual and reproductive health (SRH) services is integral to reducing maternal mortality and upholding women's bodily autonomy. Women's autonomy in sexual and reproductive health is the right to independently make decisions about their body, sexuality, and reproductive functions—core tenants of the right to privacy, equality, and bodily integrity (1, 2). However, many women still lack the autonomy to make their own decisions around their own SRH due to a myriad of factors including their socioeconomic status, age, education, husband's education, employment status and lack of financial ownership (3). This lack of autonomy and ability to access affordable, high-quality, and respectful SRH services can drastically impact a woman's life and their enjoyment of their full human rights (1).

Modern contraceptive use is also critical to reducing maternal mortality (4, 5). Low modern contraceptive use is inversely correlated with maternal mortality and unsafe abortion (6, 7). Chad had a maternal mortality ratio (MMR) of 1,063 maternal deaths per 100,000 live births in 2020, and the WHO estimated that a Chadian 15-year-old had a 1 in 15 chance of dying due to maternal causes throughout her life—the highest lifetime risk in the world (7).

To address these critical issues, the Government of Chad has committed to increasing contraceptive prevalence from 8.1%–20% by 2030 (8), reflecting continued political will stemming back to the passage of the Reproductive Health 006 law in 2002 that guarantees an individual's right to reproductive health regardless of age or marital status (9). Despite these efforts, the number of Chadian women who have access to and consistently use modern contraceptives is alarmingly low. For example, in the 2019 Multiple Indicators Cluster Survey (MICS), 8.6% of married women aged 15–49 in Guéra province, where our study took place, used a modern contraceptive method in 2019, slightly higher than the 6.7% national contraceptive prevalence (10). Married and unmarried women of reproductive age in Guéra had, respectively, unmet need for contraception of 31.0% and 64.5% (10).

Low contraceptive use in Chad can be attributed to a multitude of factors, ranging from health systems challenges to individual, religious, and cultural norms. The national health system faces significant strain due to scarce numbers of qualified health personnel to address common health issues, leading to substandard quality of care and diminished trust in health care services (11). These health system issues are chronic due in part to Chad's limited resources, with only 3.1% of the national GDP allocated to the health sector in 2016 (11). Chadian social and religious norms also significantly curtail women's decision-making power, which impedes their ability to independently make choices about health care and modern contraception (12).

The International Rescue Committee (IRC) collaborated with the Chadian Ministry of Public Health (MOH) and the Association Tchadienne pour le Bien-Etre Familial (ASTBEF), the local member agency of the International Planned Parenthood Federation, to implement the Protection, Gender and Health (ProGeSan) program in 2020. The ProGeSan program goal was to increase access to quality maternal, newborn, infant, adolescent and sexual and reproductive health services, including response to gender-based violence, in 15 health centers in three districts of Guéra province and five refugee camps in Wadi-Fira province. ProGeSan aimed to increase the demand for these services through improving social empowerment of Chadian women of reproductive age, a key enabling factor of contraceptive use and uptake of health services in sub-Saharan African countries (13). Columbia University's Reproductive Health Access, Information and Services in Emergencies (RAISE) Initiative and the University of N'Djamena collaborated on a research to action component of the ProGeSan program. This study aimed to understand the barriers and facilitators of women's use of and decision-making about modern contraception during ProGeSan which is critical to reducing the unmet need for contraception.

## Materials and methods

This mixed-methods study was conducted in three districts of Guéra province. The study included a longitudinal survey of women and focus group discussions with male and female users and non-users of modern contraceptives. In-depth interviews were also conducted with midwives, community health workers (CHWs), and community leaders.

### Longitudinal study

The sampling frame for the survey included women aged 15–40 years old who were currently pregnant or who had delivered a baby in the previous year living in villages within 10 km of the 15 health centers supported by ProGeSan. Using MOH population estimates for the villages, 27 villages were selected using probability proportional to size. Lists were stratified by age group (15–19 years and 20 years and older); women were randomly selected from these lists in each village. Adolescents were oversampled to ensure recruitment of at least 100 out of a total sample size of 450. Participants were interviewed three times to track changes in SRH service utilization: in June 2021, November 2021 and October 2022. The first interview was conducted with 459 women, and 364 women (79.3%) completed all three interview cycles.

The survey questionnaire was developed in French, adapted from existing tools of the *Demographic and Health Surveys* and those previously used by RAISE. The questionnaire covered participants' knowledge and use or non-use of sexual and reproductive health services, household decision-making, self-efficacy and attitudes towards intimate partner violence. Questionnaires were programmed on tablets using CommCare (14). Female interviewers were recruited from the host community and trained for 8 days, during which they practiced interviewing techniques and agreed upon how they would translate the questions into Chadian Arabic. The training also covered best practices for interviewing, SRH topics, and research ethics.

CHWs for each village and the community leader assisted the team to find selected women. At Cycle 1, if a selected woman was not recognized or no longer lived in the village, the interviewer replaced her with a woman from a list of randomly selected alternates. No replacements were made in later cycles. If the woman was absent, the teams made an additional return visit to the village to complete interviews. Each evening, data were uploaded to CommCare's servers from the tablets.

### Qualitative methods

After the third cycle, 8 focus group discussions (FGDs) were conducted, two each with female non-users of contraception, female contraceptive users, male non-users of contraceptives, and husbands of contraceptive users. A semi-structured FGD guide asked about the barriers and facilitators of contraceptive use, with whom people in the community discussed contraception, contraceptive knowledge and preferences. The discussions with non-users focused on social norms that guide decision-making around contraceptive use and asked them to complete a participatory ranking exercise during which they collaboratively ranked their own responses in terms of order of importance, providing comprehensive data on both group and individual perceptions.

In-depth interviews were conducted with one midwife, one CHW and one religious or community leader in or near each of four supported health facilities. Semi-structured interview guides addressed the contraceptive services or awareness-raising activities they provide, people's reasons for using or not using contraceptives, difficulties encountered in raising awareness of contraceptives among women and their husbands. The research team recruited four experienced qualitative researchers (two male, two female), and trained them for two days on the study and data collection procedures.

### Analysis

Survey data were downloaded from CommCare, and subsequently imported into SPSS (v28) for cleaning and analysis. Analysis was limited to women who completed three interviews. Descriptive analyses were run, including comparisons of sociodemographic characteristics between adolescent and adult respondents or changes from Cycle 1 to Cycle 3. Significance was determined with *p*-values < .05. McNemar's tests were used to compare dichotomous variables across time. Missing data (less than 1% for all variables) were excluded from analysis. A sensitivity analysis was used to compare the demographics of women who completed three cycles with those who dropped out.

Interviews and FGDs were audio-recorded with participant consent, transcribed, translated into French and reviewed for accuracy. US and Chadian researchers used thematic analysis to identify the main patterns and themes from the transcripts that contribute to understanding of the survey findings The research team then collaboratively reviewed and refined these themes to ensure they accurately captured participant perspectives. Further analysis was conducted by a research assistant at Columbia University, who mapped quotes onto a thematic matrix or chart in Excel for systematic organization and interpretation of the data.

### Ethical considerations

The study and its associated consent procedures received ethical approval from the Institutional Review Board of Columbia University (AAAT0905) and the Direction de la Recherche, de l'Innovation of the Ministry of Higher Education (017/PR/MESRI/DGM/DTGESRI/DRI/21) in Chad. All participants provided verbal informed consent. For participants aged 15–17, parental consent requirements were waived due to this study being classified as presenting minimal risk, with verbal informed consent sought from the participants themselves, following the same procedures as for those age 18 and over. Survey respondents were identified with a code number, and the paper lists used to find the women were destroyed on completion of data collection. The file linking the code number and identifying information was saved on a password-protected computer.

To ensure confidentiality, all FGDs and in-depth interviews were conducted in private rooms or outdoor spaces away from other people. Any names mentioned by participants in the interviews or focus groups were redacted during transcription. Only members of the research team had access to these data.

## Results

The survey analysis is limited to the 364 women aged 15–40 who completed three interviews. In-depth interviews were completed with four midwives and four CHWs. Eight focus group discussions were conducted, two groups with each of the following categories: female contraceptive users, male contraceptive users (or whose wives used), female non-users and male non-users of contraception. Each group had 6–9 participants.

### Participant sociodemographic characteristics

About a third of our survey participants (30.5%) were adolescents aged 15–19 years old, 40.6% were 20–29 years old, and 26.9% were 30–40 years old (Table 1). Nearly all participants were Muslim (90.1% of adolescents and 100% of adults). Adolescents were more educated than adult women with 36.0% having at least some secondary education compared to 8.7% of adult women. Almost half (43.5%) of adult women had no formal education compared to 16.2% of adolescents.

**Table 1:** Sociodemographic characteristics.

Characteristics	Total	Adolescents, 15–19 years	Adults, 20–40 years	*p*-value
	(*N* = 364) %(n)	(*N* = 111)	(*N* = 253)	
Age				
15–19 years	30.5% (111)			
20–29 years	42.6% (155)			
30–40 years	26.9% (98)			
Religion				
Muslim	97.0% (353)	90.1% (100)	100.0% (253)	<.001
Christian	3.0% (11)	9.9% (11)	0.0% (0)	
Educational Attainment				<.001
No formal education	35.2% (128)	16.2% (18)	43.5% (110)	
Some or completed Primary school	47.8% (174)	47.7% (53)	47.8% (121)	
Some or completed Secondary school or higher	17.0% (62)	36.0% (40)	8.7% (22)	
Marital status				
Married or cohabiting	91.8% (334)	75.7% (84)	98.8% (250)	<.001
In a polygamous marriage	43.0% (142)	33.3% (26)	46.0% (116)	.07
Age at first marriage mean, standard deviation (SD)	15.1 (2.4)	14.5 (1.9)	15.2 (2.1)	.007
10–15	37.6% (126)	47.6% (40)	34.3% (86)	.04
15–17	49.6% (166)	45.2% (38)	51.0% (128)	
18 and older	12.8% (43)	7.1% (6)	14.7% (37)	
Husband education				.23
No formal education	52.0% (173)	45.2% (38)	54.2% (135)	
Some or completed primary school	18.0% (60)	19.0% (16)	17.7% (44)	
Some or completed secondary school or higher	24.6% (82)	32.1% (27)	22.1% (55)	
Don’t know	5.4% (18)	3.6% (3)	6.0% (15)	
Ever been pregnant	91.5% (333)	72.1% (80)	100.0% (253)	<.001
Mean number of lifetime pregnancies (SD)	5.8 (3.4)	2.4 (1.6)	7.0 (3.0)	<.001

Nearly all adult women (98.8%) were married during the study, compared to 75.7% of adolescents. The vast majority of participants first married before the age of 18, at a mean age of 14.5 years among adolescents and 15.2 years among adults. Most women's husbands (88.7%) were more than five years older than them. The husbands of just over half of participants (55.1%) had no formal education, while 19.0% had at least some primary education, and 25.9% had some secondary education or higher. Fertility was high in this population with adult women reporting a mean 7.0 pregnancies compared to 2.4 pregnancies among adolescents. Beyond these sociodemographic characteristics, few differences were found between adolescent and adult women's results; therefore, further results are reported for all participants together. In our comparison of participants who completed three cycles and those lost to follow-up, we found no sociodemographic differences, with one exception. Women whose husbands had at least some education were more likely to complete three cycles (*p* = .02).

### Contraceptive knowledge

Knowledge of all modern contraceptive methods improved from Cycle 1 to Cycle 3 (Table 2). While injectables were the best known method, the greatest increase in knowledge was for condoms and tubal ligation. IUDs and vasectomy were the least known.

**Table 2:** Contraceptive knowledge.

Contraceptive method	Cycle 1 (*n* = 364)	Cycle 3 (*n* = 364)	*p*-value
Ever heard of tubal ligation	28.3% (103)	54.4% (198)	<.001
Ever heard of vasectomy	6.0% (22)	11.3% (41)	.02
Ever heard of intra-uterine device (IUD)	11.0% (40)	18.1% (66)	.006
Ever heard of implant	72.0% (262)	89.8% (327)	<.001
Ever heard of injectable	92.9% (338)	97.5% (355)	.002
Ever heard of pill	67.0% (244)	84.6% (308)	<.001
Ever heard of condoms	42.9% (156)	69.2% (252)	<.001
Ever heard of emergency contraception	19.5% (71)	27.7% (101)	.008

This high level of awareness of injectables was also reflected in the qualitative data, with contraceptive non-users demonstrating strong knowledge of injectables during the focus groups. Non-users ranked injectables as the most effective and acceptable contraceptive, followed by oral contraceptive pills, while long-acting reversible contraceptives (LARCs) ranked last if at all mentioned. Some female and male non-users of contraceptives appeared to view contraceptives as synonymous with injectables and used these terms interchangeably.

For us, if a man here wants to limit births, he can simply ask his wife to go get the injections. We don't know much about these medications or methods. (Male non-users 1)

Despite relatively high levels of awareness of some methods, contraceptive non-users described misconceptions about contraceptive methods, how they work, and their side effects. Many expressed fears that IUDs and implants would cause sterility or disappear in the body, or that they would be stuck with the method if the health centers were to close. These misconceptions appeared to be widespread, as they were mentioned by women, men, midwives, and CHWs.

The least accepted [method] is that of 2 years and 5 years [implants]. Women here do not often use this; they say it will cause sterility; it will disappear in the body. (Female non-users 2)

IUDs and implants, they're [women in the community] not very interested in them. When I've asked, they ask won't it bother them afterwards? And won't it bother the husband during sex? Won't it disappear one day? Some of them say that if you insert it [implant], it disappears. (Midwife 4)

### Source of information on contraception

Survey participants who reported using a modern contraceptive method at least once during the study and those who reported no contraceptive use reported similar sources of information about contraception in the past year at Cycle 3 (Table 3). They most often mentioned the health center (98.1% of users, 79.6% of non-users), followed by CHWs (47.2% of users, 40.0% of non-users) and the women's peers—family, friends, and husbands (24.5% of users, 35.0% of non-users). Few participants reported the radio (12.4%), community or religious leaders (4.9%), or school (2.3%) as information sources.

**Table 3:** Source of information on contraceptives over the past 12 months, Cycle 3.

Information source	Women who reported modern contraceptive use during the study	Women who reported no modern contraceptive use during the study	Total
	(*N* = 106) % (*n*)	(*N* = 240) % (*n*)	*N* = 346
Health center	98.1% (104)	79.6% (191)	85.3% (295)
Community health workers (CHW)	47.2% (50)	40.0% (96)	42.2% (146)
Family, friends, husband	24.5% (26)	35.0% (84)	31.8% (110)
Radio	17.9% (19)	10.0% (24)	12.4% (43)
Community or religious leader	8.5% (9)	3.3% (8)	4.9% (17)
School	0.9% (1)	2.9% (7)	2.3% (8)

The FGDs yielded similar results, with female contraceptive users and non-users saying they received information from midwives at the health center as well as from their female peers. Contraceptive users reported that conversations with female friends who had used a method helped them decide to use themselves. Several participants noted that hearing from other women who had used the injectable that it was “good” helped assuage their worries and convinced them to choose it.

“I heard about the method at the hospital, and I myself decided to use it. The women who used it before me said this one for 3 months [injectable] is good, and since I've been using it, I have no worries.” (Female contraceptive users 8).

Male FGD participants also said that conversations with their friends, family members, and neighbors influenced their decision to use contraceptives. The male non-users ranked friends as the top people with whom they discussed contraceptive decision-making, while their wives did not even make the list. Women were aware of their husbands' friends' influence, with one contraceptive user saying she would recommend women whose husbands won't allow them to use contraception to ask one of his close friends to “*advocate with her husband with well-founded arguments so he agrees she can use the injectable*” *(Female contraceptive users 8).* Similarly, male users in one group thought they should tell their peers about the benefits of contraceptives.

“When you know the merits of something, you can tell your brother so that he benefits too. Just as it benefited you, it can also benefit your brother.” (Male contraceptive users 3)

For example, it was me who told my wife. Also, we talk about contraceptive methods even to the neighbors. Women who use them also do the same with their neighbors. It creates a kind of neighborhood awareness. (Male contraceptive users 3)

Similar to the survey results, women reported CHWs as another common information source. CHWs' awareness raising included traveling door to door or neighborhood to neighborhood with illustrative flip charts providing information about contraceptives. Men noted that these campaigns, which occurred at mosques or during weddings or other ceremonies, were their main information source. One man expressed wanting to hear more from the imams at the mosques, since imams hold significant authority and influence in their community. Several men noted that the use of flip charts and awareness raising materials by CHWs was particularly helpful to their understanding the benefits of contraception and ultimately deciding to use a method.

“Sometimes if a woman tells [the CHW] that her husband refuses, the CHW will come and find the husband to personally explain the merits of using contraceptive methods so that he accepts.” (Male contraceptive users 7).

Yes, you also have to use images to illustrate and enable people to better understand the different contraceptive methods and their benefits for the reproductive health of mother and child. (Male contraceptive users 3)

### Contraceptive use

At Cycle 3, 20.5% of survey participants reported currently using a modern contraceptive (Table 4). The most used method at Cycle 3 was injectables (76.1%), followed by implants (17.9%). Overall, 30.6% of survey participants reported current use of modern contraceptive method during at least one of the three cycles, with most reporting birth spacing as the reason (84.3%). The majority (93.4%) of contraceptive users noted that their husbands were aware of their contraceptive use, and 95.3% expressed that their husbands approved. Multiple ways in which contraceptive use helped them were cited, with the most common being that their children are better off and healthier (80.8%), that they don't have to worry about getting pregnant (54.8%), and that they were less tired or felt stronger and healthier (30.8%).

**Table 4:** Modern contraceptive use.

Variable	Total *N* = 346
Reported current modern contraceptive use at Cycle 3	20.5% (67)
Currently using Injectables	76.1% (51)
Currently using Implant	17.9% (12)
Currently using Pills	6.0% (4)
Reported current modern contraceptive use at least once during the study period	30.6% (106)
	(*N* = 106)
Primary reason for contraceptive use	
Birth spacing or don't want more children	84.3% (91)
Other reasons	15.7% (17)
Husband involvement	
Husband is aware of her contraceptive use	93.4% (99)
Husband approves of her contraceptive use	95.3% (101)
How contraceptive use has helped her	
Children are healthy, better off	80.8% (84)
I have no worries about getting pregnant	54.8% (57)
I am less tired/less weak/stronger/healthy	30.8% (32)
I have more time for myself	16.3% (17)
Small families are better off	14.4% (15)
Economic benefits	13.5% (14)

Female and male contraceptive users in the FGDs described similar reasons for using contraception, including the need for the woman to recover between pregnancies and give their babies the chance to grow before having another. Others referred to poverty and the lack of resources to feed and educate their children. Women described the incredible amount of work they had working in the fields and managing the household, while husbands mentioned that when the woman was suffering, the entire household suffered too.

For me, it's poverty that pushes me to take [contraception]. We have a lot of children, and we don't have enough to feed them, and the husband just doesn’t care. (Female contraceptive users 4)

A woman who hasn't finished breastfeeding, if she takes on another pregnancy, this is a burden for her in the upbringing and care of her baby, but also for her husband. …In reality, when the mother is suffering, you, her husband, are also suffering. You've got too much on your plate too. You have to buy medicines, pay for milk, etc. Seeing all this, we understood that there are too many problems and that we have to find solutions. Also, the woman herself won't be in great shape. Every year, she gives birth, which will tire her out a lot. She'll be exhausted. She'll lose weight. (Male contraceptive users 3)

Contraceptive users in the FGDs also described reasons why they preferred injectables, including fewer side effects and that they were easy to use. They described oral contraceptives as causing menstrual irregularity, heavy periods, and nausea, and that women were likely to forget to take them. They also indicated a general dislike of the idea of a method like LARCs being physically placed inside the body.

Me and my wife, we discussed using the pills. She refused because she might forget or misplace the pills, or sometimes people say that they cause nausea. As for the [implant], she told me that she doesn't want something that's always in her arm. She told me she prefers the injectable, and I told her that anyway, these medications all play the same role. (Male contraceptive users 7)

My wife uses the [implant]. There’s nothing else to do, because you get the [implant] inserted, and then you rest until it finishes. But the pills, you have to take every day which is tiring and sometimes she might forget. (Male contraceptive users 7)

Midwife perceptions of the suitability of specific methods for certain demographics of women also played a role in which methods they counselled women to use. Some midwives noted that they would not recommend LARCs for unmarried women or adolescents, to whom they would recommend condoms. All four midwives expressed some hesitation with IUD insertion due to their limited practical experience with them. Some described needing additional training in this skill, or lacking supplies.

The IUD in particular is not something we can administer here. We don't have it, so we don't do it here, and if I were asked to do it, I'd feel less comfortable doing something I haven't done before. (Midwife 1)

### Barriers to contraceptive use

Among women who reported no contraceptive use during the study, the majority reported fertility-related reasons, including partner absence or infrequent sex (73.2%) or a desire to get pregnant (33.8%) as reasons for non-use (Table 5).

**Table 5:** Reasons for not using contraception among women who reported no modern contraceptive use during the study (*n* = 228).

Reasons for non-use	% (*n*)
Fertility-related reasons^a^	73.2% (167)
Desire for pregnancy	33.8% (77)
Up to God/fatalistic	42.1% (96)
Opposition to use^b^	28.1% (64)
Reasons related to methods^c^	12.7% (29)
Lack knowledge (methods, source)^d^	9.6% (22)
Lack access^e^	3.1% (7)
Other reasons^f^	7.5% (17)

^a^ Fertility-related reasons include those who are unmarried or whose husband is absent; are not having sex or infrequent sex; are having difficulty getting pregnant; are postpartum amenorrheic or breastfeeding.

^b^ Opposition to use includes those who oppose contraceptive use or heard that contraception is bad for her; whose husband opposes; who report it being against their religion.

^c^ Method-related reasons include those who fear side effects; say that the method is inconvenient or difficult to use; report health-related reasons or say that contraception doesn't work.

^d^ Lack of knowledge includes those who know no method; know no source of methods; lack information or don't have enough information about contraception; or say they have never heard of contraception.

^e^ Lack of access includes those who say services are too far or too expensive; her preferred method is not available; the services are not confidential; the providers have bad attitudes.

^f^ Other includes those who want to wait for a particular number of births before using; said she is not yet ready to use; or that she doesn't need contraception.

Among focus group participants, stigma was the most frequently mentioned barrier to contraceptive use. They expressed concern that people in their community would talk negatively about them if they were to use contraceptives. Male non-users of contraceptives frequently labelled women using contraception as sex workers or cheating on their husband. Adolescent girls were mentioned as particularly afraid of the social stigma.

Girls are afraid to go to the health center to ask….They say that people will start saying they're looking for men or marriage. (Midwife 3)

These young people are afraid because our very facility is exposed. We [the health facility] don't have a fence, we don't have a building where a young person can come in secret. Maybe the day we have a building and the center is fenced off, we hope they'll come. But it’s not easy, they'll be in need—we don't know—but they're afraid to come and get a contraceptive method. (Midwife 1)

Two CHWs noted that stigma in some communities made raising awareness in these areas very difficult, and they often faced backlash due to contraception being viewed as a “white” or foreign practice that wasn't needed. In one FGD, men described contraception as a foreign thing to kill their wives and children.

It's true that our main job is to educate the community on contraceptive methods, but the major difficulties are linked to the reluctance of the population. This attitude doesn't make our job easy at all. We often wait for people to tell us that our injectable isn't any good, that it's a thing for white people, and that they don't want it. It really weakens our awareness-raising. (CHW 1)

Perceptions that fertility is up to God was reported by 42.1% of contraceptive non-users and a common theme in the focus groups. A male participant articulated his reasoning for not using contraceptives:

It's Allah who gives children, it's also him who stops giving. If he gives, we accept, if he doesn't, alhamdulillah. (Male non-users 1)

As in the survey, fear of side effects came up only rarely in the focus groups although as mentioned above, many described misconceptions about some methods. One CHW noted that they had heard people say they didn't use condoms because they reduced sexual pleasure, while others mentioned that contraceptive methods disrupt menstrual cycles and bother women.

We don't use contraceptive methods, because women say that the injectables disrupt their menstrual cycle. Some even say they lose a lot of blood. (Male non-users 1)

They say the injection is no good because when they menstruate…the periods that don't come during the time they use a contraceptive method accumulate and then come all at once in quantity when they stop using the injectable. That's why they don't like it. (CHW 1)

One midwife mentioned stockouts of contraceptive methods influencing availability and uptake of contraceptives, specifically oral contraceptives, and a CHW noted that condoms were rarely available at facilities. However, supply issues or lack of access did not arise in the focus groups as a reason for non-use.

More than a quarter (28.1%) of contraceptive non-users reported that opposition—by their husbands, themselves or their religion—was one of their reasons for non-use. Several CHWs cited women's opposition to modern contraceptives—due largely to misconceptions around contraceptives being either morally wrong or physically harmful — as a significant challenge for them. In such instances, CHWs described making multiple home visits to discuss the benefits of contraception, which they said often resulted in women eventually deciding to use contraception. While “Koranic prohibition” was occasionally mentioned by FGD participants, the female non-user FGD that mentioned it ranked it as the least important of ten reasons cited for women's non-use. Among survey participants who reported opposition to use, husband's opposition was the most commonly reported source of opposition, a phenomenon that emerged in the focus groups as well.

### Husbands' role in contraceptive use

Both men and women described the substantial role husbands played in a couple's contraceptive decision-making as either a major facilitator or barrier to their wife's ability to use contraceptives. Nearly all women in the FGDs noted they first discussed the use of contraceptives with their husbands before seeking contraceptive services. Men's opinions on contraceptives were deemed to be the final deciding factor.

Many men do not accept that their wives use the injectable to space births. When you ask him and he refuses, you must obey his decision because he is your husband. (Female contraceptive users 8)

Husbands oppose their wives' [contraceptive use] because he is the master of the family and she has no choice but to obey. If the wife does this [uses a method], her husband will divorce her because of this contraceptive method. (CHW 4)

Yes, the wife and husband must discuss the use of contraceptive methods before going to the health center. If the couple doesn't discuss, they can't use. If the wife decides to use contraception, she must have her husband's permission. If it's the husband who wants his wife to use it, he has to explain the reasons to convince his wife, so discussion is important. (Male non-users 5)

A few midwives and all four CHWs described that husband accompaniment of their wives to obtain contraceptives was important for them to provide a method. If husbands were not available, CHWs would call them to confirm their approval, or the woman could come with her brother or brother-in-law. They described how a woman using contraception without husband permission was risky and could potentially result in community or husband aggression towards contraceptive providers, or trouble within the marriage, thus making them reluctant to provide it.

“If she comes alone to take the injection, we won't give it to her. She must be accompanied by her husband. But if she comes without her husband, we won't give it to her. There’s no such thing.” (CHW 2)

If she comes alone, the doctor may not believe her and refuse to give her the method she asked for…Without the husband, the doctor won't give anything because women are sometimes complicated. (Male contraceptive users 3)

Midwives had a more nuanced view of the need for husband consent. All four midwives were aware of the 006 Reproductive Health law, which explicitly gives women the right to seek contraception on their own. However, two of four midwives expressed some concern about providing contraceptives without husband permission out of fear of him causing problems in the community.

Two midwives expressed steadfast support of women who sought contraception without their husbands, and reported feeling that their provision of contraceptives was protected by 006 Law.

If she comes without her husband, if it's her wish, she chooses her method and I'll provide it. I'll do it because law 006 authorizes me to provide these contraceptive methods to women of childbearing age…. I'm also a woman, I put myself first in my sister's shoes. Given the situation we live in, if she comes to me, I first share her pain. I share with her what she's feeling and if she chooses a method, I don't see the problem in providing it. I feel calm, at ease with these women who come openly to me asking for this service. (Midwife 1)

But if [her husband] refuses [to use FP] and the woman comes and asks me to rescue her, I'll do it….With law 006, which protects the midwife, I'm not afraid of that…. I'll do it because I'm a woman like her. I know the pain she feels, and that's why I take this risk to help her. (Midwife 2)

## Discussion

Although the increases in participants' knowledge and high modern contraceptive use at the end cannot be attributed solely to the ProGeSan program, they suggest that the program may have successfully increased contraceptive awareness and use, given that contraceptive prevalence in our study was twice as high as that reported in this population in the 2019 MICS (10).

While the limited use of LARCs in our study could be explained solely by women's personal preferences, the qualitative data revealed widespread misconceptions around LARCs. CHWs should seek to actively dismantle these misconceptions in the community. Other programs in Chad have found success with mobilizing influential men and women in the community who use these methods as “satisfied users” willing to speak about their IUD use, coupled with using mass media and interpersonal channels to describe the benefits of IUDs, like an immediate return to fertility upon removal (15). Reducing the misconceptions and fear around LARCs may increase uptake of modern contraceptives and diversify the method mix.

Providers should also seek to address these misconceptions while delivering balanced contraceptive counseling to clients. The low client load for IUDs contributes to midwives' lack of confidence in their IUD insertion skills, making them less likely to offer IUDs as an option (16). It is important to also strengthen midwives' support systems, including through supportive supervision, coaching and refresher training, to ensure women's access to the full range of contraceptive methods available (15, 17).

Given the preference for injectables, the program should encourage the MOH to introduce the self-injectable contraceptive (DMPA-SC), which has been piloted in Chad but not broadly rolled out, and is an increasingly popular choice for community-based service delivery (18). A 2019 systematic review concluded that DMPA-SC yields comparable or improved contraceptive continuation rates compared to provider-administered injectables, without significant increases in pregnancy rates or safety concerns (19). Evidence from other countries also found that DMPA-SC was highly accepted, with 80% of study participants reporting confidence in self-injection after receiving training, and 97% describing it as easy after 3 months (20). As with any method, DMPA-SC will not appeal to everyone, though evidence supports that increasing diversity of method mix leads to increased contraceptive use (21). Given the important role of CHWs in this community, training CHWs in community-based distribution of DMPA-SC and other methods, including condoms, should be explored to reduce access barriers and provide an option for a discrete contraceptive that does not require going to the health facility, potentially reaching more first-time contraceptive users and adolescent girls (22).

Despite midwives' awareness of Chad's 2002 Reproductive Health Law 006 protecting women's right to contraceptive access, some still expressed discomfort providing contraceptives to women without explicit husband consent. Given that CHWs and midwives are from the communities they work in, some expressed some fear of backlash from husbands and the community. Similar provider restrictions have been found in other countries based on marital status (23–25). Midwives mentioned that it was rare for unmarried adolescents to seek contraception due to community stigma, and one mentioned they would only recommend condoms to adolescents and unmarried women, a common example of provider bias which can limit women's agency and choice around contraception (24). This perception of contraception as only appropriate for certain women under certain conditions represents a key barrier to women's ability to uphold their bodily autonomy and participate in decision-making around their own health and fertility. Values clarification and attitude transformation (VCAT) workshops should be held with midwives and CHWs to examine and reflect upon the underlying beliefs behind these attitudes and their impact on women and girls (26). Future interventions should continue to closely collaborate with health workers to promote non-discrimination and equity in service provision through supportive and non-judgmental addressing of provider bias, identifying provider champions to positively influence their coworkers, proactively clarifying the negative consequences of provider restrictions, and using comprehensive social and behavior change approaches (24). Programs should provide stigma reduction interventions to CHWs, addressing their own misconceptions and stigmatizing attitudes, while also training them on how to respond to community stigma and misconceptions (26). Other successful interventions have shifted the focus from blaming CHWs and other providers for their behavior to using a more positive framing that focuses on empowerment and increasing their accountability and autonomy (27).

Similar to findings elsewhere, male partner opposition to contraception or desire for more children was a common reason for women's non-use of contraception across many contexts (28, 29). A review of factors influencing contraceptive decision-making in crisis-impacted areas in sub-Saharan Africa found that nine of twelve studies determined that male influence was the strongest factor in women's choice around using contraception (30). Men in our study also echoed the importance of their own role in their wives' contraceptive use, a finding supported by evidence from across many contexts (31).

For these reasons, expanding male engagement is crucial to increasing contraceptive use. One multi-country study found that men in Kenya who had participated in at least one community activity, and men in Senegal who had seen family planning messaging on television or heard a religious leader positively discuss contraception were more likely to use modern contraception (28). Programming should be expanded beyond general community awareness campaigns to include tailored male-friendly approaches like using male ambassadors or champions, working with community leaders, identifying and targeting hotspots in the community where men congregate, and using mass media strategies (28, 32). Promoting healthy couple communication, which is associated with increased contraceptive use and gender equality, should also be explored (33). For example, programming in Niger effectively engaged married adolescent couples through a combination of CHW home visits and small gender-segregated group discussions, resulting in increased modern contraception use and decreased intimate partner violence (34).

Few survey participants reported receiving contraceptive information in the last year from community or religious leaders. CHWs occasionally visited mosques to raise awareness, typically during ceremonies. However, both CHWs and male FGD participants highlighted the potential they saw in increasing collaboration with imams and other community leaders to spread positive messages about contraceptive use due to their respected role in the community. Research from several African, predominantly Muslim, countries finds that religious leaders can be either a key facilitator or a barrier to contraceptive use (35, 36). In Burkina Faso, Muslim leaders were more accepting of contraceptive use when it was framed as promoting the health of the mother and child (36). Programming to increase contraceptive demand in Senegal found that exposure to a religious leader speaking positively about contraception was correlated with higher odds of modern contraceptive use (28). This evidence suggests that further engagement with religious and community leaders in this area through reframing contraception as promoting the health of families and its alignment with Islamic faith may be a promising approach to shifting social norms and increasing contraceptive uptake.

### Limitations

Although efforts were made to reduce attrition, some survey participants were lost to follow up at subsequent cycles. Women whose husbands had no formal education were more likely to be lost to follow up, though other socio-demographic characteristics were the same as among participants. A multi-level analysis of the factors associated with modern contraceptive use among married women in sub-Saharan Africa found that women whose husbands had at least primary education had higher odds of using modern contraception, suggesting that our contraceptive use estimates may be overestimated (37). Some individuals presented conflicting statements at different cycles in the study. Efforts were made to clarify these conflicting statements with women; when this was not possible, those variables were determined to be missing. While the researchers tried to use the same interviewers at each survey cycle, they were not always available. In such situations, the research team recruited and trained new interviewers, which may have introduced variability in interviews. As survey respondents were self-reporting their attitudes and behavior, some could have misreported their true answers due to social desirability bias.

## Conclusions

Women in our study demonstrated high levels of knowledge of some contraceptive methods, though slightly less than a third of the sample used contraception at any point in the study period, with the majority of participants opting to use injectables. Women reported that their husbands' role in decision-making, including husbands' acceptance and accompaniment of their wives to seek contraception, was critical to their ability to use contraception. The most reported reasons for non-use were views that fertility is up to God or opposition by husband, religion, or oneself. Community stigma and common misconceptions about some methods, particularly LARCs, were also significant barriers to contraceptive use. More diverse and tailored stigma reduction interventions should be implemented in the community, including interventions to engage men, couples, and religious and community leaders, to facilitate awareness and use of modern contraception.

## Data Availability

The raw data supporting the conclusions of this article will be made available by the authors, without undue reservation, to any qualified researcher upon reasonable request.
